# An Oil-Free Picodrop Bioassay Platform for Synthetic Biology

**DOI:** 10.1038/s41598-018-25577-4

**Published:** 2018-05-21

**Authors:** Christian A. Siltanen, Russell H. Cole, Sean Poust, Lawrence Chao, Jabus Tyerman, Benjamin Kaufmann-Malaga, Jeff Ubersax, Zev J. Gartner, Adam R. Abate

**Affiliations:** 1 Department of Bioengineering and Therapeutic Sciences, University of California, San Francisco, San Francisco, California, USA; 2 Amyris, Inc. Emeryville, California, USA; 3 Department of Pharmaceutical Chemistry, University of California, San Francisco, San Francisco, California, USA; 4 Chan Zuckerberg Biohub, San Francisco, California, USA; 5 Delv Bio, Sacramento, California, USA

## Abstract

Droplet microfluidics enables massively-parallel analysis of single cells, biomolecules, and chemicals, making it valuable for high-throughput screens. However, many hydrophobic analytes are soluble in carrier oils, preventing their quantitative analysis with the method. We apply Printed Droplet Microfluidics to construct defined reactions with chemicals and cells incubated under air on an open array. The method interfaces with most bioanalytical tools and retains hydrophobic compounds in compartmentalized reactors, allowing their quantitation.

## Introduction

Synthetic biologists engineer organisms to produce high-value compounds, including drugs, biofuels, and chemical building blocks^1–5^. This often depends on a design-build-test cycle in which libraries of genetic variants are constructed and tested for their ability to synthesize the desired compound. While molecular techniques can generate libraries of enormous diversity^6–8^, each variant must be tested for the phenotype of interest, necessitating costly and time-consuming screening^9–11^. Indeed, the costs and time of screening are often principal barriers to the commercial success of a synthetic biology product.

The gold standard in high-throughput screening is robotic liquid handling in microliter well plates^12^. This approach has several features that make it useful across biology^13^. It is a standardized and universal hardware platform, allowing it to be repurposed for different applications by reprogramming. It delivers quantitative reagent volumes, allowing reproducible protocol execution. The open array interfaces with most bioanalytical tools, like fluorescence, mass and optical spectroscopy, and sequencing. Nevertheless, the size of synthetic biology screens make even this high throughput approach costly^14^, with the major bottlenecks being time and reagent consumption. For example, to screen a modest million-member library would take over a week^15^, and cost tens of thousands of dollars in pipette tips. To drive down screening cost and increase success, assay volumes must be reduced to the smallest screenable unit of genetic libraries: Single cells.

By shrinking assays to picoliters, droplet microfluidics allows cost-effective screening of a scale previously unthinkable^16^, making it useful for applications from evolving catalysts to automating cell transformation, culture, and recombinant protein expression^15,17^. Nevertheless, droplet microfluidics has significant constraints that limit its broad implementation. Cells are compartmentalized in oil, precluding assays with hydrophobic compounds, like lipid biofuels and many drugs, that exit droplets. In addition, the approach can be inflexible, requiring a custom-built device for each application, and that may have limited capability. A strategy that combined the throughput of droplet microfluidics with the flexibility of microliter robotics would thus address a major barrier in synthetic biology strain optimization.

In this paper, we describe a reprogrammable nanoliter bioassay platform for synthetic biology. The approach builds upon our recently described Printed Droplet Microfluidics (PDM) platform, a new form of droplet microfluidics that constructs reactions on an ordered array rather than in a disordered emulsion pack^18^. The array functions analogously to microliter well plates – but at one-thousandth the scale – and uses a “microfluidic robot” for deterministic and reprogrammable reagent and cell dispensing. However, first generation PDM confined drops under an oil reservoir and did not provide long term culture of viable cells. Here, we describe advances that enable label-free colony detection, extended cell culture, and methods for retention and screening of hydrophobic secreted bioanalytes. We demonstrate this with *S*. *cerevisiae* engineered to produce *trans*-β-farnesene, a sesquiterpene of the mevalonate and deoxyxylulose-5-phosphate pathways that can be derivatized into numerous high-value products^1,19^. Screening for this and other hydrophobics has not been previously possible with droplet microfluidics, due to their solubility in emulsion oil^20–22^. By adapting PDM to compartmentalize reactions under air, rather than oil, farnesene molecules remain partitioned with the cells responsible for their production, allowing direct measurements of product concentration for screening. PDM thus provides a powerful platform for nanoliter assays combining the throughput and low reagent usage of droplet microfluidics with the flexibility and control of robotic fluid handling on arrays.

## Results and Discussion

**Figure 1:**
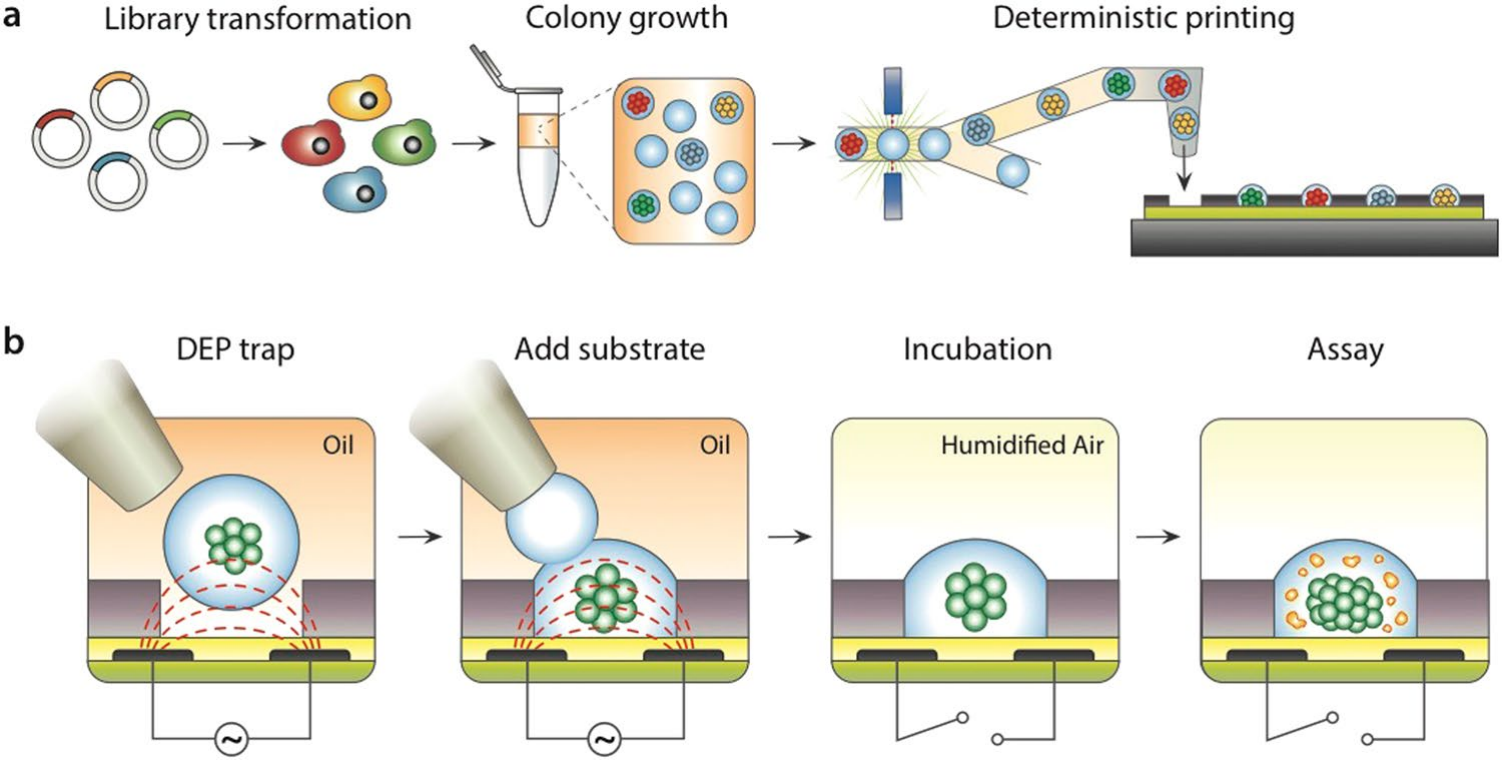
Synthetic yeast library printing and screening scheme. (**a**) Single yeast cells are encapsulated and incubated in picoliter droplets to generate a suspension of isogenic colonies. Droplets are interrogated in a microfluidic droplet sorter and dispensed to a microwell array based on user-defined gating parameters. (**b**) The microwell array substrate is registered to microelectrodes, creating dielectrophoretic traps that pull droplets into nanoliter wells, where multiple droplets can be combined. After printing isogenic colonies, each well can be supplemented with droplets containing assay reagents or fresh culture media. Printing oil is removed and the cells cultured under humidified air for growth and subsequent assay.

Our approach is designed to detect small differences in farnesene production between similar strains of yeast. We begin by encapsulating single *S*. *cerevisiae* cells at limiting dilution^23^ from an engineered library in 300 pL droplets, ensuring that each is associated with a single genotype and phenotype (Fig. 1a). To increase assay sensitivity, we expand single cells into colonies by pre-culturing in the droplets for 24 hr^24^. To avoid confounding farnesene yield and growth phenotypes, we select droplets for downstream analysis with similar colony density. Cell density is assayed by optical density by flowing droplet cultures past a laser/transmission detector, followed by dielectrophoretic (DEP) sorting and printing of similar cultures^25^. Selected droplets are ejected from a nozzle and guided into nanoliter wells *via* DEP traps patterned on the array surface (Fig. 1b)^26^. The traps destabilize droplets, allowing several to be added to each well; this allows cells, media, and assay reagents to be added in a fully programmed and unique sequence for each reactor, something not possible with merger or picoinjection in conventional droplet microfluidics^27–29^. After printing, the colonies are supplemented with fresh media and, when necessary, encapsulated in hydrogel by printing drops of crosslinkable polymers. The carrier oil above the array is then replaced with humidified air (methods) for incubation and analysis (Fig. 1b).

**Figure 2:**
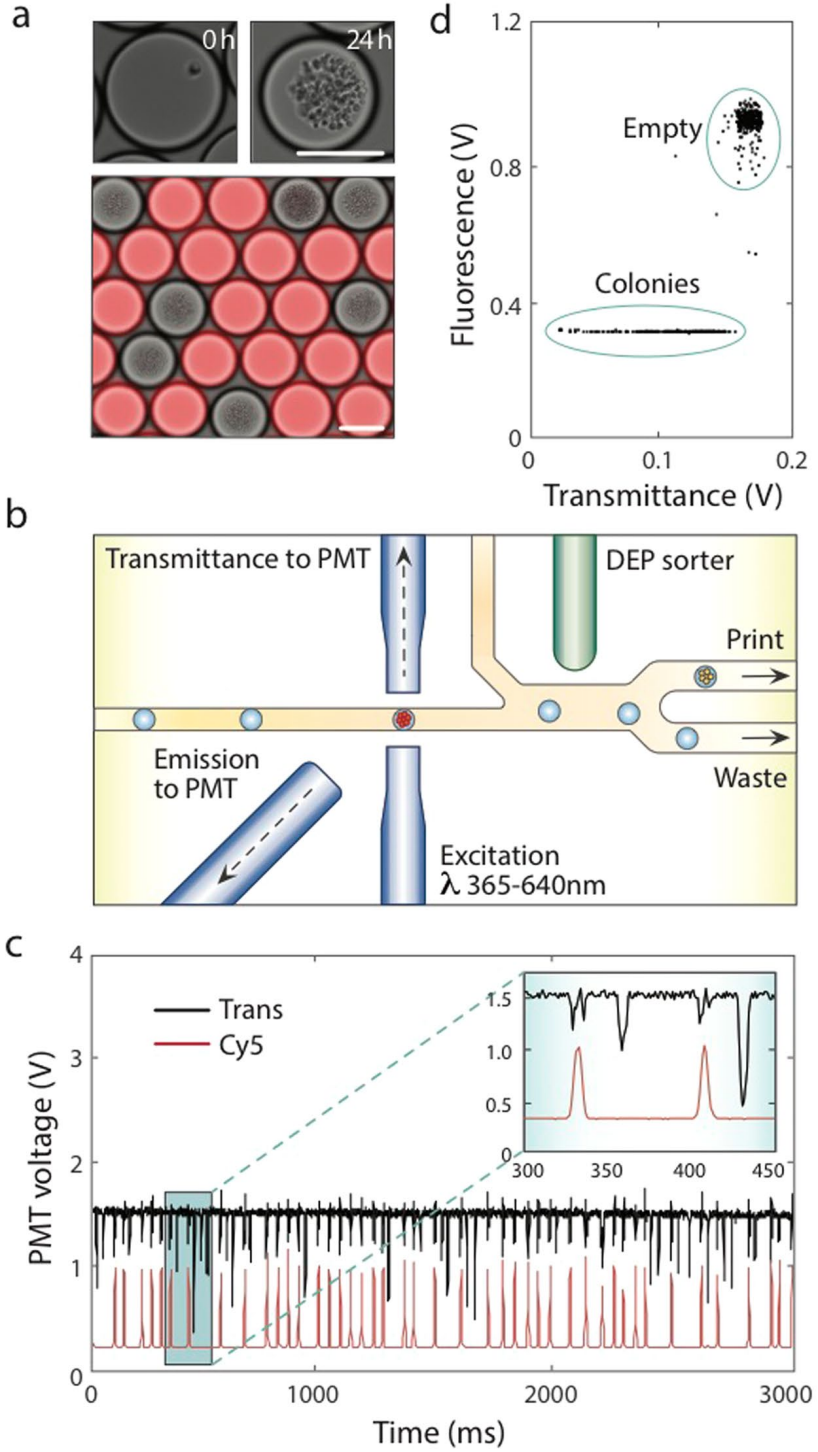
Isogenic colony expansion and detection using fluorescence and absorbance activated droplet sorting (FAADS). (**a**) To illustrate FAADS analysis, yeast cells are encapsulated in unstained droplets and mixed with a separate emulsion of fresh media (red). (**b**) The mixed emulsion is injected into the PDM chip where droplets flow through an interrogation channel consisting of a light source-coupled fiber and two detection fibers registered to PMTs. (**c**) PMT voltage time traces are measured to identify signal peaks in droplet fluorescence and absorbance, where colonies cause a density-dependent decrease in transmittance. (**d**) Droplet fluorescence is plotted versus time-averaged transmittance, and the clusters gated for dispensing. Scale bars = 75 μm.

To demonstrate this scheme on our PDM platform, we created emulsions consisting of empty (Cy5 labeled) and colony-containing droplets (Fig. 2a). Labeling different drop types with fluorescent dyes gives them a discernable optical signature required for identification and sorting. Even for genetically identical cells, we found that colonies grow to different densities, which may be due to differences in seeding cell state^30^. To reduce variation in the downstream assay, we thus inoculate the arrayed nanoliter cultures with only colonies of similar cell density. This required a method for label-free colony detection, which we accomplished by measuring optical density (OD)^25^ (Fig. 2b and c) within the print-head and dispensing only colonies of similar size. Empty droplets are tightly clustered in the fluorescence versus transmittance plot, while colony-containing droplets vary in transmittance due to differences in cell density (Fig. 2d). By strictly gating for high OD droplets, we thus dispense only colonies of similar size. The ability to discard abnormal colonies prior to analysis is not possible with robotic liquid handling in well plates, and key to accurate analyte production measurement.

**Figure 3:**
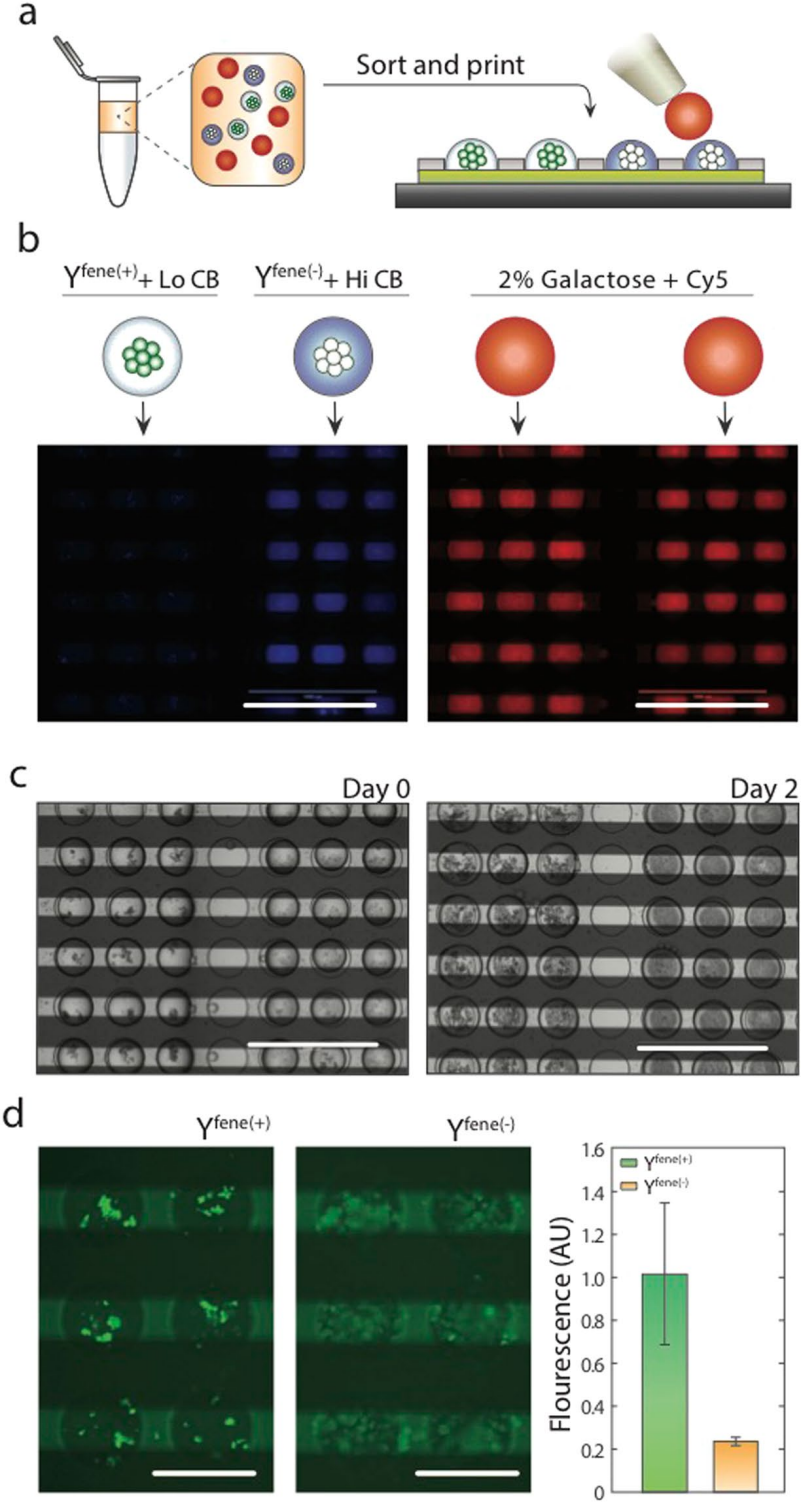
On-chip culture and farnesene bioassay. (**a**) A mixed emulsion of engineered isogenic yeast colonies is loaded into the PDM chip and printed. (**b**) Y^*fene*(+)^ colonies (unlabeled) are printed next to Y^*fene*(−)^ colonies (blue dye labeled). In a subsequent step, all wells are supplemented with fresh media (red dye labeled) to initiate farnesene production. (**c**) Yeast colonies proliferate in culture under humidified air. (**d**) Printed yeast colonies are encased in PEG hydrogel by adding droplets containing crosslinkable polymer. Secreted farnesene accumulates in the gelled wells, allowing a miscible Nile Red solution to be added while maintaining compartmentalization, showing a 5-fold increase in Y^*fene*(+)^ signal (p < 0.002). Scale bars = 1 mm in (**b**) and (**c**), and 400 μm in (**d**).

To illustrate the power of PDM for synthetic biology, we use it to measure farnesene production in two strains of yeast: an engineered variant (Y^*fene*(+)^) used in industrial production, and a wildtype that does not produce the molecule (Y^*fene*(−)^). The farnesene producing Y^*fene*(+)^ strain is derived from the amorphadiene-producing engineered *S*.*cerevisiae* S288C strain Y293 through three modifications^31^; (i) The amorphadiene synthase on the plasmid pAM426 is replaced with an *A*.*annua* farnesene synthase; (ii) ERG12 under galactose regulation (*P*_*GAL1*_) was introduced at GAL80; and (iii) random mutagenesis with ethyl methanesulfonate and selection by Nile Red fluorescence staining. As an internal control in the PDM assay, we pre-labeled droplets with genotype indexing dyes (3.3 μM and 33 μM Cascade Blue in drops containing Y^*fene*(−)^ and Y^*fene*(+)^, respectively). We print the colonies to adjacent substrate regions and, in a subsequent step, add fresh media labeled with a secondary reference dye (Cy5) (Fig. 3a). After oil removal, colonies are cultured, growing to fill their wells (Fig. 3b).

As farnesene is produced, it accumulates as lipid droplets that can be quantified via fluorescent dyes such as Nile Red. However, lipophilic stains are generally used as cytotoxic end-point assays, and must be delivered to each microwell without the use of carrier oils which may wash away the farnesene product. To introduce the Nile Red dye while still preserving co-localization of farnesene droplets with their cognate cell colony, we developed a method to physically retain the target within a hydrogel mesh. During the initial colony inoculation in microwells, we printed droplets containing PEG-diacrylate and 4-arm PEG-thiol polymers which, upon mixing, crosslink *via* a Michael-addition reaction to form a flexible hydrogel. This gelation chemistry was chosen for its biocompatibility and kinetics under physiologic conditions, which has been previously validated to support viability and proliferation of yeast and even delicate mammalian cells^32,33^. Furthermore, this PEG hydrogel formulation has been previously validated with “virtual microfluidics” for whole genome amplification and sequencing of single encapsulated microbial cells^34^, a critical downstream step in strain optimization design-build-test cycles. Indeed, we observed colony growth for both tested strains of PEG-encapsulated cells. We then stained for farnesene synthesis by covering the entire array in an aqueous solution of Nile Red, and observed ~5-fold increased fluorescence signal for Y^*fene*(+)^ relative to wildtype (Fig. 3c). The background wildtype Y^*fene*(−)^ fluorescence corresponds to absence of farnesene, and results from staining of hydrophobic compounds naturally present in cells, like lipid membranes. These results are consistent with bulk liquid phase assays and illustrate the potential of PDM for analyte production screening with single cell seeded nanoliter cultures (Figure S2).

PDM overcomes longstanding barriers in droplet microfluidics, like the inability to screen for hydrophobic compounds or register droplets on an array that can be monitored over time. PDM also allows reactors to be constructed deterministically with specific combinations of reagents added to each well. Such control is key to the success and universality of liquid handling robots. By providing a thousand-fold reduction in well volumes, however, PDM dramatically increases the scale of screens possible. Moreover, the unique ability of PDM to isolate, culture, and analyze single cells affords new assay opportunities. Combined, these properties should enhance synthetic biology screens by making them more affordable and increasing the potential to discover rare, valuable variants in large libraries.

## Methods

### Microarray substrate fabrication

We designed our microarray geometry to minimize the electric field strength required for both DEP droplet trapping and droplet merging, while allowing us to maintain droplet position after removing the applied field and oil phase for culture. We thus empirically tested the effects of varying the electrode gap (50–250 μm), microwell diameter (150–300 μm) and depth (25–100 μm), and applied AC power (5–50 kHz, 100–1000 V). We found that a 175 μm electrode gap and 300 μm diameter wells provided the most reproducible printing at all applied voltages, while well depths greater than 50 μm performed less reliably.

Electrode arrays were patterned with photolithography. A 200 Å layer of chromium is sputter deposited onto glass slides (LGA Thin Films, Santa Clara, CA), then wet etched using ma-P 1215 positive photoresist (Microchem, Westborough, MA) as a photopatterned protective layer. The electrode array consists of 200 interdigitated chromium strips connected to bipolar electrical contact pads. A 10 μm thin film of PDMS is spin-coated onto the electrode array to create an insulating dielectric layer and, after exposing the PDMS thin film to oxygen plasma, the substrate is coated with a 50 μm layer of SU-8 photoresist to pattern microwell arrays. The SU-8 is processed according to the manufacturer’s instructions to create a grid of 300 μm diameter wells (400 μm pitch) centered between electrodes. During printing experiments, the substrate is submerged under Fluorinert FC-40 oil (Sigma Aldrich, St. Louis, MO) in a petri dish and connected to 300 V, 20 kHz AC power supplied by a function generator and amplified by a high voltage amplifier (Model 2220, Trek). Electrical connections are made with SEM conductive tape (Ted Pella, Redding, CA).

### PDM printhead fabrication

The PDM chips are fabricated in poly(dimethylsiloxane) (PDMS) by soft lithography. A two-layer SU-8 negative master is patterned on silicon wafers with 75 μm height flow channels and sorting channels, and 200 μm height guide channels for insertion of optical fibers and nozzle tubing. Liquid PDMS is poured, cast, and cut from the SU-8 master and inlet ports are punched with a 0.75 mm biopsy core, prior to plasma bonding to a 50 mm × 75 mm glass slide. A 1 cm PVDF capillary tubing (200 μm OD, 75 μm ID) (Paradigm Optics, Vancouver, WA) is inserted into the nozzle channel and sealed with epoxy glue, and all channels are treated with Aquapel (Pittsburgh, PA) to render surfaces hydrophobic. The droplets used in the PDM device are generated on a separate PDMS chip, and re-injected into the PDM device for printing. Droplet generator chips consist of a 60 μm x 60 μm flow-focusing channel geometry.

### PDM chip fluidics and optical configuration

The PDM instrument was modified from our previously published design^18^. 80 μm droplets are first generated in the separate PDMS flow focusing device and collected in a solution of Novec HFE-7500 (3 M, Minneapolis, MN) stabilized with 2% v/v PEGylated fluorosurfactant (RAN Biotechnologies, Beverly, MA) in a syringe. The droplet suspension is re-injected into the PDM chip *via* syringe pumps (New Era, Farmingdale, NY) at a flow rate of 50–100 μl/h for printing. The droplet sorting fluidics also include two carrier fluid channels, consisting of HFE-7500 oil with 0.2% fluorosurfactant, each flowing at 1,500 μl/h, and a waste channel operated at negative pressure to tune the relative rates of carrier oils flowing to the printer nozzle vs. waste. Electrode channels and a “Faraday moat” are filled with a 1 M solution of salt water for sorting. The PDM chip is affixed to an XYZ micromanipulator and positioned with the print nozzle near the printer substrate mounted on a mechanical stage (MA-2000, ASI, Eugene, OR) and visualized with an epifluorescence microscope (AE31, Motic, Hong Kong). The optical configuration is modified from our previously published protocol^18^. All optical fibers (Thorlabs, Newton, NJ) are stripped, inserted into respective channels in the PDM chip, and fixed with kapton tape. The multimode excitation fiber has a cladding diameter of 125 μm and optical core diameter of 105 μm with an NA of 0.22. It is connected *via* coupling fibers to 100 mW continuous-wave lasers with wavelengths of 405 nm, 473 nm, 532 nm, and 640 nm (CNI Lasers, Changchun, China) and laser power is adjusted so that each color reaches the end of the excitation fiber at ~ 1 mW power. Emitted and transmitted light are collected by a 225 μm cladding diameter, 200 μm core diameter fiber with NA of 0.39, and a 125 μm cladding diameter, 25 μm core diameter fiber with NA of 0.1, respectively. Light collected by the emission fiber is collimated and directed through a series of dichroic mirrors to the emission detection PMTs (Thorlabs) with bandpass filters centered at 448 nm, 510 nm, and 697 nm. Transmitted light is collected and directed to a transmittance detection PMT with a bandpass filter centered at 571 nm.

### PDM operation

Droplet printing is automated through a custom LabVIEW application. Optical detection data is acquired by a field programmable gate array (FPGA, National Instruments, Austin, TX) and displayed in LabVIEW, where gates are set by the user to program the FPGA to trigger an electrical signal to the sorting electrode via a high voltage amplifier (690E-6, Trek, Lockport, NY), directing the selected droplet to the printer nozzle *via* dielectrophoresis. The position of the nozzle is rastered relative to the print substrate by moving the mechanical stage, which is controlled by the LabVIEW application.

### Yeast encapsulation, suspension culture and printing

Yeast strains and a proprietary media were supplied by Amyris, Inc (Emeryville, CA) on agar plates. For printing experiments, colonies are picked and inoculated in shaken liquid culture at 1000 rpm for 24 h at 30 °C in media supplemented with 2% Galactose. Prior to encapsulation, the cells are resuspended in fresh media containing dye labels and injected into the flow focusing chip at a concentration of 1.6e^5^-mL^−1^, corresponding to a mean number of 0.05 cells per droplet, limiting doublet rates to <0.2% based on Poisson encapsulation^23^. Y^*fene*(+)^ and Y^*fene*(−)^ cell containing drops are differentially labeled with 33 μM and 3.3 μM dextran-conjugated Cascade Blue (10 kDa, Thermo Fisher, Waltham, MA), respectively. Single cells are encapsulated in 80 μm droplets and incubated in suspension for an additional 48 h at 30 °C to reach stationary phase. The colony-laden droplet suspension is then mixed with droplets containing fresh media, labeled with 10 μM sulfo-Cy5 dye (Lumiprobe, Hallandale Beach, FL), and re-injected into the PDM chip for printing. For PDM microarray yeast culture, a print file was written to deposit one droplet containing a cell colony followed by 20 diluent droplets of fresh media to each microwell, for a total final volume of ~6 nL per well. After printing, the array is sealed in a petri dish next to a large (>5 mL) reservoir of media to provide a humidified environment. The dish is left to equilibrate for 30 min at room temperature, then printing oil is aspirated with a syringe, the electrodes are disconnected from power, and the dish is transferred to a 30 °C incubator for culture. Microwell cultures and cell growth are monitored by imaging on a fluorescence microscope (EVOS, ThermoFisher).

### PEG hydrogel encapsulation and Nile Red staining

For hydrogel encapsulation experiments, diluent droplets were prepared with either 1% w/v PEGDA (700 Da, Sigma-Aldrich) or 7% w/v 4-arm PEG-thiol (10 kDa, Laysan Bio, Arab, AL) in PBS containing 10 mM Na_2_CO_3_ (pH 7.5). Diluent droplet emulsions were re-injected into the PDM chip, where they are printed to microwells at a 1:1 ratio (10 droplets each, ~6nL total volume per well) such that the ratio of reactive acrylate and thiol groups on the PEG hydrogel precursor polymers are stoichiometrically balanced. Upon mixing in microwells, the gels crosslink in ~10 min. Upon gelation, oil is removed and the entire array is rinsed with fresh culture media, and incubated in a humidified petri dish at 30 °C. After 24 h the cells are stained for farnesene production by incubating the array with PBS containing 6.2 μM Nile Red, then rinsing with PBS. Since Nile Red stains lipids non-specifically, fluorescence intensity from each well is normalized to biomass area using brightfield thresholding with ImageJ.

### Statistical information

For Nile Red fluorescence staining quantitation, reported values represent mean  ± SD for n = 25 wells.

## Electronic supplementary material

Supplementary Information
