# Supplementary material for: An Oil-Free Picodrop Bioassay Platform for Synthetic Biology
Source: Sci Rep. 2018 May 21;8:7913. doi: 10.1038/s41598-018-25577-4 (PMC5962535; doi:10.1038/s41598-018-25577-4)
Supplement: Supplementary file 1 — Supplementary Information [file 41598_2018_25577_MOESM1_ESM.docx]

**Supplementary Information**

**An Oil-Free Picodrop Bioassay Platform for Synthetic Biology**

**Christian A. Siltanen^1^, Russell H. Cole^1^, Sean Poust^1^, Lawrence Chao^2^, Jabus Tyerman^2^, Benjamin Kaufman-Malaga^2^, Jeff Ubersax^2^, Zev J. Gartner^3,4^, Adam R. Abate^1,4^***

^1^ Department of Bioengineering and Therapeutic Sciences, University of California, San Francisco, San Francisco, California, USA

^2^ Amyris, Inc. Emeryville, California, USA

^3^ Department of Pharmaceutical Chemistry, University of California, San Francisco, San Francisco, California, USA

^4^ Chan Zuckerberg Biohub, San Francisco, California, USA

* Corresponding Author


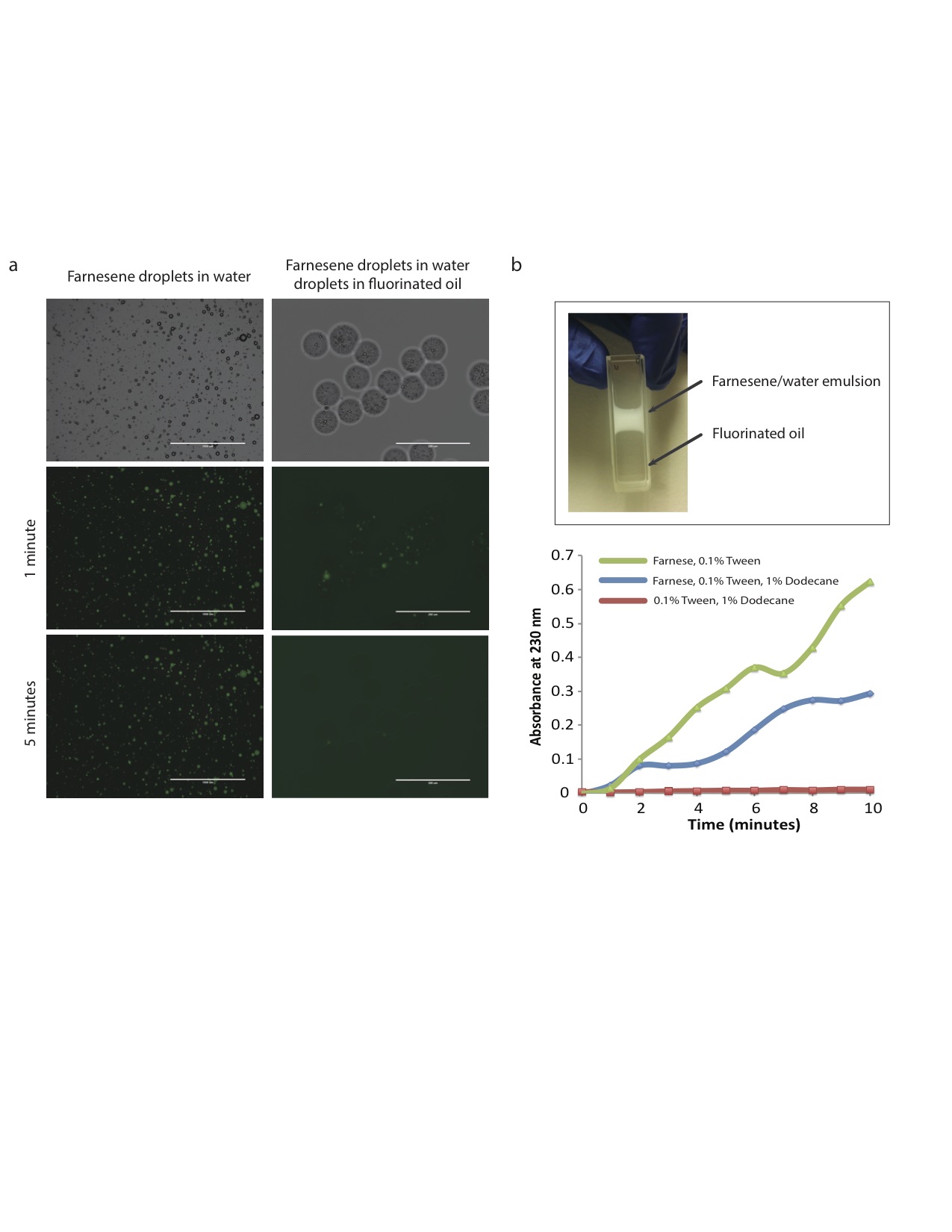


**Figure S1.** Farnesene and Nile Red rapidly partition from aqueous droplets into fluorinated oil, even without fluorosurfactant. a) A solution of PBS with 0.1% v/v Tween-20 and Nile Red is agitated with farnesene to produce stable lipid droplets (left panel). The mixture is encapsulated in HFE-7500 oil via microfluidic flow focusing. Within 5 min the aqueous droplets are nearly devoid of farnesene, which has partitioned into the carrier oil. b) UV absorbance analysis shows that farnesene partitions into fluorinated oil even in the absence of fluorosurfactant, indicating that native solubility of farnesene in oil, not micelle-mediated transport, is responsible for leakage. Scale bars = 200μm.


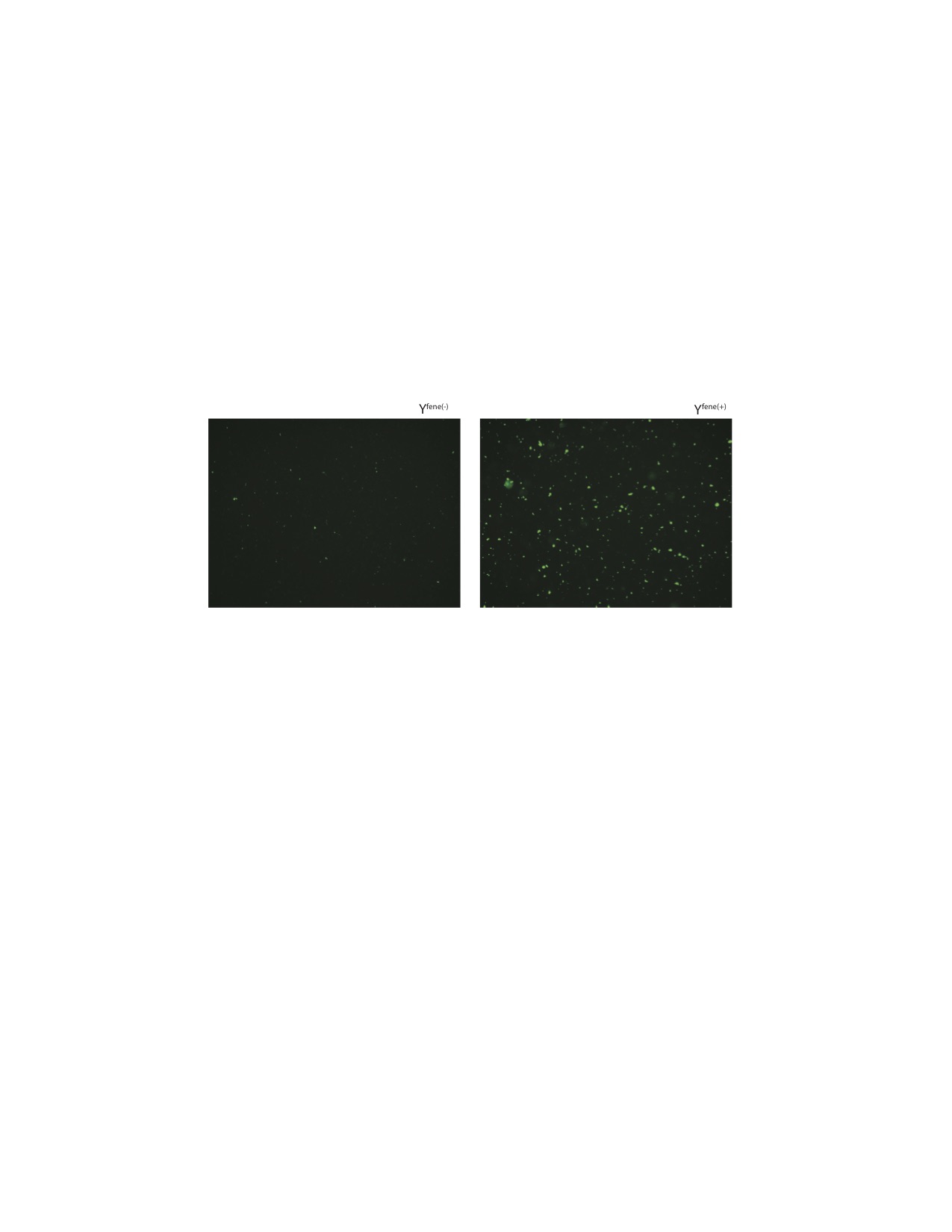


**Figure S2.**  Bulk liquid cultures of Y1151 cells fluoresce strongly with Nile Red staining, indicating lipid biosynthesis. A background signal is visible in Y17025 cells due to staining of cell biomass.
